# Motivational Interviewing Counseling to Increase Endocrine Therapy Adherence in Diverse Patients

**DOI:** 10.3390/cancers15071973

**Published:** 2023-03-25

**Authors:** Stephanie B. Wheeler, Jennifer Spencer, Sarah W. Drier, Niasha Fray, Katherine E. Reeder-Hayes

**Affiliations:** 1 Department of Health Policy and Management, University of North Carolina at Chapel Hill, Chapel Hill, NC 27599, USA; 2 Lineberger Comprehensive Cancer Center, University of North Carolina at Chapel Hill, Chapel Hill, NC 27599, USA; 3 University of Texas Austin Dell Medical School, Austin, TX 78713, USA; 4 Division of Hematology and Oncology, University of North Carolina at Chapel Hill, Chapel Hill, NC 27599, USA

**Keywords:** breast cancer, endocrine therapy, behavioral intervention, motivational interviewing, health disparities

## Abstract

**Simple Summary:**

Up to ten years of oral endocrine therapy can help prevent breast cancer recurrence and death in approximately 70% of patients diagnosed with hormone-receptor positive breast cancer for whom it is prescribed, but long-term adherence is low, particularly among Black women and those younger than age 50. Because the barriers to adherence are multifaceted, in this study, we designed and pilot-tested a flexible, evidence-based counseling intervention to help diverse women set breast health goals and identify and overcome the barriers to adherence. The results demonstrated a strong interest in the intervention, willingness to complete follow-up counseling sessions, high satisfaction with the intervention, and high adherence to endocrine therapy at twelve months, especially among Black participants, providing promising signals concerning the benefits of supportive behavioral counseling and motivating future work in this area.

**Abstract:**

Background: Oral endocrine therapy (ET) is an inexpensive and effective therapy for hormone receptor-positive (HR+) breast cancer that prevents recurrence but relies upon long-term adherence for up to ten years. More than 80% of breast cancer patients have an HR+ phenotype and are candidates for ET, but approximately half discontinue or become non-adherent by five years. ET underuse is more prevalent in Black and young (<50 yrs) women, which may contribute to outcome disparities in these groups. The objective of this study was to evaluate the feasibility, acceptability, and utility of a patient-centered counseling intervention to enhance ET adherence, with a focus on the needs of Black and younger women. Methods: We conducted a single-arm pilot study of a twelve-month motivational interviewing (MI) intervention consisting of five MI counseling sessions, an interactive workbook, a resource guide, and an educational video developed and revised with iterative patient and clinician input. The eligible participants were >18 years old, English speaking, and with stage I–III HR+ breast cancer. Participants were recruited across a large academic medical center and four community sites. Feasibility and acceptability were assessed by measures of participant recruitment, retention, session participation, and patient-reported satisfaction. ET adherence at 12 months was assessed by self-report and medication event monitoring system (MEMS) caps using a continuous measure of the proportion of days covered (PDC) as well as a dichotomous measure of the optimal adherence, defined as >80% PDC. Results: Forty-two women initiated the intervention, of whom thirty-five participants (83%) completed outcome assessments at 12 months, including thirteen Black and twenty-two non-Black participants. The average participant age was 54.8 years (range: 25–73). Overall, 97% completed at least three MI sessions and 83% completed at least four sessions. Participant retention and satisfaction were high, particularly among Black women. Self-reported adherence at 12 months was 88% overall (100% in Black women and 81% in non-Black women). The majority of women also achieved 80% of days adherent using MEMS caps, with a greater adherence in Black women. Conclusions: This study demonstrates the feasibility, acceptability, and early promise of the effectiveness of an MI counseling-based intervention to promote ET adherence and prevent breast cancer recurrence in diverse populations.

## 1. Introduction

Oral endocrine therapy (ET) is a highly effective medication that is used to prevent breast cancer recurrence and mortality among people with hormone receptor-positive (HR+) cancers when taken regularly for five to ten years. However, data from multiple contexts and settings have shown that ET adherence is sub-optimal, with up to 50% of people not taking ET as prescribed at five years [1,2]. Non-adherence is more prevalent in Black patients [3] and people younger than 50 years old [4], and prior research has demonstrated that the barriers to adherence are multifaceted and distributed differentially in these sub-populations [1]. Patient-level factors associated with ET non-adherence in observational studies include the poor management of ET-related side effects, the lack of belief in treatment efficacy, less access to ET refills [5], poor communication, and the lack of shared decision-making with providers.

These barriers to ET adherence may be amenable to intervention, but behavioral interventions to improve ET utilization among breast cancer survivors are lacking, in part because the reasons for ET non-adherence are complex and person-specific. Motivational Interviewing (MI) is an effective, patient-centered, yet directive counseling approach based in a strong health behavior theory that has been shown to improve medication adherence and other health-promoting behaviors in non-cancer populations [6,7,8,9,10,11,12,13,14,15]. Its highly individualized nature makes it particularly effective for increasing medication adherence in diverse populations and, hence, MI is an especially promising strategy for addressing the complex drivers of ET use. Because it is unclear how well an evidence-based MI intervention will translate to ET medication-taking behavior in patients with breast cancer, rigorous adaptation as well as feasibility and acceptability testing of MI-based interventions designed to support the use of ET are needed.

Drawing upon the Information–Motivation–Behavioral Skills Model [16,17], intermediate psychosocial variables that predict behavior include the following: self-efficacy, motivation, attitudes, and knowledge and skills leading to action. The many factors that influence a person’s motivation and self-efficacy to take ET include regimen characteristics (such as frequency of doses and side effects) as well as health beliefs, health status, knowledge, and risk perceptions. ET medication self-efficacy and motivation are also influenced by provider- and social/structural-level factors (e.g., clear and respectful patient-provider communication, medication cost and access, and ease of accessibility of medication support) [5]. As a result, successful interventions to address ET medication-taking behavior must (1) identify factors that inhibit and enhance individual motivation and self-efficacy; (2) raise patients’ awareness of their relative level of motivation and self-efficacy; and (3) help patients and providers develop strategies to overcome barriers and enhance facilitators [18,19]. By identifying the precursors to motivation and efficacy at the individual level, MI is well-suited to promote the readiness to change across racially diverse and different age populations. Additionally, because MI is individualized, it can be modified to address cultural sensitivity, contextual nuances, and person-specific needs.

To design a culturally sensitive intervention that addresses the barriers and leverage facilitators to ET utilization among diverse patients with cancer, including Black and young patients, and that can also be implemented in a variety of oncology care settings, we developed and tested the feasibility, acceptability, and utility of a primarily remotely delivered MI-based counseling intervention to support ET adherence in academic and community practices. To our knowledge, this is the first MI-based oral medication adherence intervention developed and piloted in patients with cancer.

## 2. Materials and Methods

### 2.1. Study Overview

**Figure A1:**
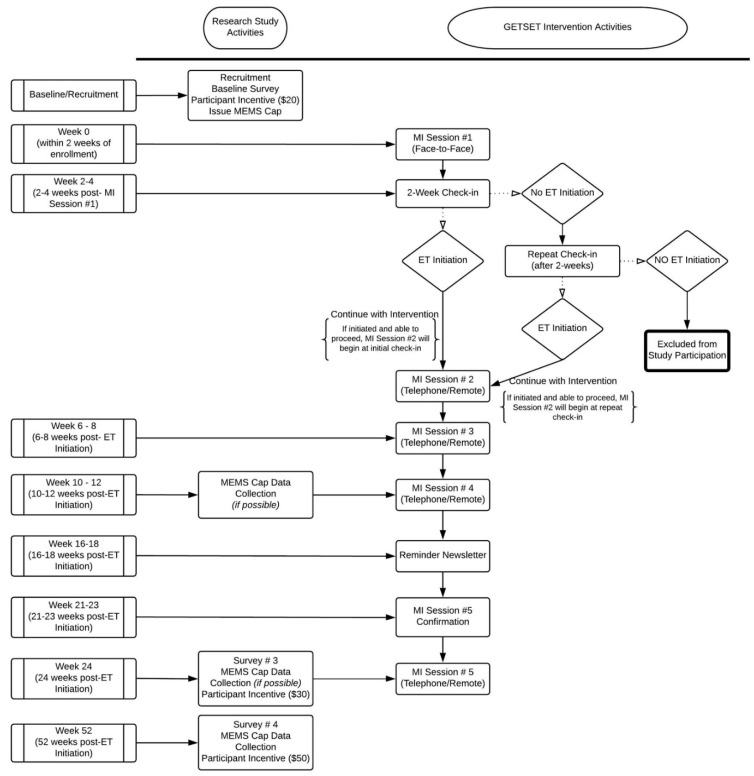
Study flow and timeline of activities.

We conducted a single-arm pilot study assessing the feasibility, acceptability, and potential utility of a twelve-month MI intervention for increasing ET adherence. Participants were recruited across five oncology clinics within the University of North Carolina’s Cancer Care Network, including one large academic medical center and four affiliated community clinics. Patients provided permission for abstraction of cancer history data from medical records and completed a baseline survey at the first study visit. Follow-up surveys were sent electronically (or by mail if requested) at six and twelve months after the first study visit. Patients received USD 20 for completion of the baseline survey, USD 30 for completion of the six-month survey, and USD 50 for completion of the twelve-month survey. Patients were provided with an electronic medication event monitoring System (MEMS) cap and bottle (Aardex Group, Belgium) and were asked to use this device to store their ET medication for the duration of the study. A full guide to intervention activities and study timeline is shown in Figure A1.

### 2.2. Participants

Eligible participants were >18 years of age, able to speak and read English, and had a stage I–III endocrine receptor-positive breast cancer. Patients were approached when possible by a member of the study team at the appointment in which they received their ET prescription or within 6 weeks of ET prescription (thus, they were all new users of ET). Patients were eligible to begin the intervention once all surgery, radiation therapy, and chemotherapy treatments were complete. The study was specifically designed to recruit at least one-third Black participants and one-third participants younger than 50, and recruitment materials (images, language, and design) were developed with input from these groups accordingly. All participants identified as women. Study activities were approved by the University of North Carolina’s Institutional Review Board (IRB #13-0736) and informed consent was obtained.

### 2.3. Motivational Interviewing Intervention

Our intervention, called GETSET (Guiding Endocrine Therapy Success through Empowerment and Teamwork), was developed using information from ET patient qualitative studies led by our team, as well as methods adapted from previous MI-based medication adherence interventions for the HIV population [19,20,21]. The intervention included five counselor-led sessions; the first was delivered in person (baseline) and lasted 60–90 min, on average, and the remaining four were delivered over the phone (at approximately 3, 6, 9, 12, and 24 weeks after ET initiation) and lasted 30–60 min, on average. The structure of each session was highly adaptable to elicit and reflect specific patient concerns. Intervention activities were structured to identify ET-related knowledge and motivations for ET adherence, facilitate goal setting, and build self-efficacy for behavior changes (see Table A1). Each participant was assigned to one of two MI-trained counselors (SWD or NF) who conducted all five sessions. With their MI counselors, participants were guided through activities that included person-centered goal-setting, identifying support networks and strategies, anticipating current and future barriers to meeting their goals, brainstorming approaches to overcome barriers, and continually adapting the plan as new challenges emerged (e.g., burdensome side effects, cost and transportation difficulties, lack of motivation, competing demands, etc.) A patient workbook was also provided, which included a guide to both local and online resources for breast cancer survivors and worksheets designed to support the content covered in each session and facilitate note-keeping. We used diverse patient and clinician focus groups to pre-test key content, graphic design, formatting, and acceptability of our intervention materials.

### 2.4. Participant Characteristics at Baseline

We collected patient sociodemographic characteristics and treatment history at baseline. Sociodemographic measures were self-reported and they included age, marital status, educational attainment, annual household income, number of dependents in household, and insurance status and type. Treatment history and type of ET prescribed were obtained from medical record abstractions.

To assess baseline medication self-efficacy regarding medication-taking behavior, women completed the Medication Understanding and Use Self-Efficacy (MUSE) scale [22]. This validated scale includes eight items assessing self-reported efficacy of understanding, seeking information about, and using a specific prescription medication. MUSE was developed for patients of varying literacy levels and socioeconomic backgrounds and it includes questions such as “It is easy for me to take my medicine on time” and “It is easy for me to get all the information I need about my medication”. The baseline composite MUSE scores reported in GETSET were compared to prior population averages in the literature.

ET medication-taking confidence was assessed using three statements on a four-point Likert scale: *(I am confident I can take ET as prescribed by my doctor, I am confident I can stay on ET for 5 years,* and *I am worried I may have to stop ET earlier than 5 years).*

### 2.5. Outcomes and Analysis

As the study was designed to address unique adherence barriers faced by Black women in particular, all study outcomes were analyzed overall and by race (Black vs. non-Black).

Our primary study outcomes were feasibility and acceptability of the MI intervention. Feasibility was measured by participation, specifically, the number of completed MI sessions, with a target of at least 80% of patients completing four out of five MI sessions; however, we also report percent completing at least three and percent completing all five sessions. We also tracked the percent of participants who used the MEMs cap successfully, including regular use of the cap while taking medication, and return of cap data to the study team for data download at 12 months. Acceptability of the intervention was measured through a patient survey at the completion of the study. Patients were asked, using a five-point Likert scale, to report on their overall experience with GETSET, whether they felt the program helped them to stay on their ET meds, and whether the program was a good fit for their life. Participants were also asked about the acceptability of the GETSET workbook, using the MEMS, and the frequency and scheduling of counseling visits.

Our secondary outcome measures were ET adherence using MEMS and self-reported data. To account for the initial visit in which the MEMS cap was provided to the participant, the MEMS reporting window began after two consecutive days of use and continued for 365 days or until the participant returned the MEMS to the study coordinator. A percent of days covered (PDC) by ET medication was calculated by summing the number of days in which the MEMS device was opened and dividing by the total number of days in the reporting window. Participants were also asked to self-report adherence in the past 14 days using a two-item measure used previously [1]. We considered participants with a PDC of 80% (or for self-report, missing no more than 3 doses in the past two weeks) to be adherent.

## 3. Results

### 3.1. Study Enrollment

**Figure 1:**
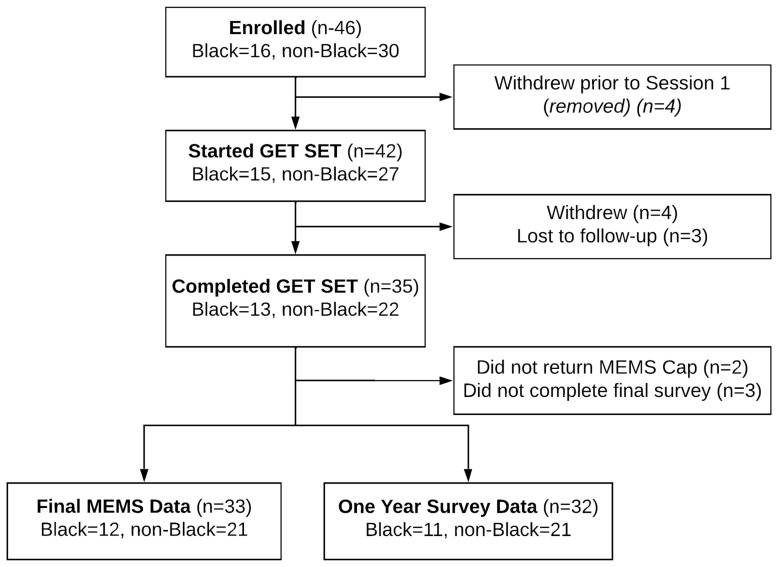
GETSET participant enrollment and study completion diagram.

A total of 46 women consented to the GETSET study (Figure 1). Of these, four did not schedule their first session or withdrew prior to initiating the intervention. Of the 42 women who started the GETSET intervention, 4 withdrew and another 3 were lost to follow-up. A total of 35 participants (83%) completed 12 months of follow-up for the intervention; of these, 33 provided MEMS data at 12 months, and 32 completed the 12-month follow-up survey.

### 3.2. Participant Characteristics

Among the participants completing 12 months of follow-up, 13 (37%) participants were Black and 22 (63%) were non-Black (Table 1). The average participant age was 54.8 years. The majority of participants were married or living with a partner (69%), although partnership was much more common among non-Black participants (95%) than Black participants (23%). A total of 74% of participants had at least a four-year college degree (including 62% of Black and 82% of non-Black participants) and 40% reported an annual household incomes greater than USD 100,000 (23% of Black; 50% of non-Black). The majority of participants were privately insured (62% Black; 82% non-Black).

### 3.3. Baseline Medication Confidence and Self-Efficacy

**Figure 2:**
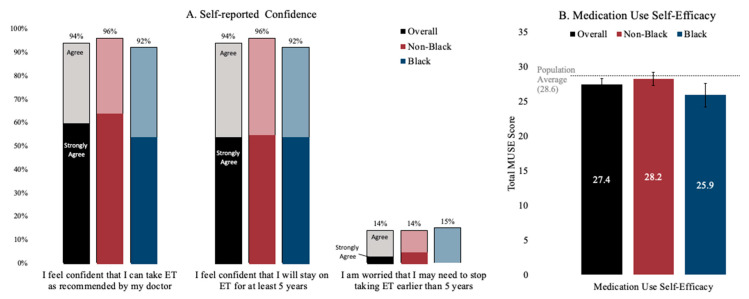
Baseline ET medication-taking confidence (**Panel A**) and self-efficacy with medication use (**Panel B**). Notes: Confidence was self-reported using a four-point Likert scale. Medication Use Self-Efficacy (MUSE) is presented as a composite score, with bars representing 95% confidence intervals.

At baseline, participants were confident about their ability to take ET as recommended (Figure 2), with an overall 94% (92% Black; 96% non-Black) *agreeing* or *strongly agreeing* that they were confident that they could take ET as recommended. Slightly more than half of the participants *strongly agreed* that they were confident in taking ET as recommended (52% Black; 64% non-Black). An overall of 14% of participants (15% Black; 13% non-Black) *agreed* or *strongly agreed* that they were worried about stopping ET earlier than five years. The MUSE medication self-efficacy scores were within the range of the previously reported averages in other works in the literature (28.6), with Black women reporting an average score of 25.9 (sd: 4.4) compared to non-Black women reporting an average score of 28.2 (sd: 3.5).

### 3.4. Primary Outcome: Feasibility and Acceptability

Among the women who completed 12 months of follow-up (N = 35), 34 (97%) completed at least three MI sessions and 29 (83%) completed at least four sessions (Table A2), whereas 19 (45%) of women completed all five sessions. The vast majority completing 12 months of follow-up was also able to contribute MEMS data at twelve months (94%), with only one participant declining use of the MEMS and a second losing the device before returning it to the study team. Among all the participants who initiated the intervention, including those who were lost to follow-up or withdrew (N = 42), 81% completed at least three MI sessions, 69% completed at least four sessions, and 45% completed all five sessions.

**Figure 3:**
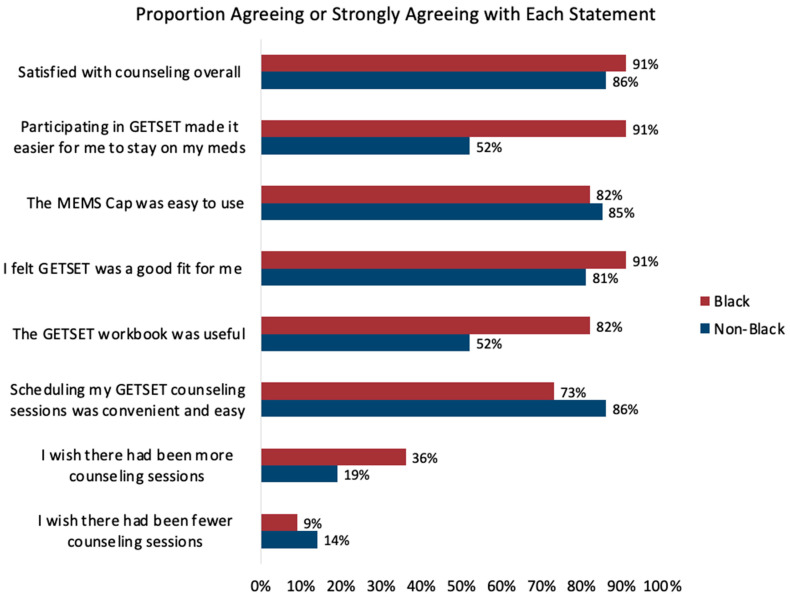
GETSET feasibility and acceptability outcomes by race.

Participants completing 12 months of follow-up generally reported a positive experience with the intervention, with 88% (91% Black; 86% non-Black) reporting satisfaction with counseling overall, and 84% (91% Black; 81% non-Black) reporting that the program was a good fit (Figure 3). Importantly, two out of three participants felt that GETSET made it easier for them to stay on their medications, including 91% of Black participants and 52% of non-Black participants. Use of the MEMS cap (84%), the workbook (65%), and the scheduling of counseling (77%) were also generally acceptable to all participants. Participants generally agreed that the number of sessions was appropriate, although 25% would have preferred more sessions and 12% would have preferred fewer sessions.

### 3.5. Secondary Outcome: ET Adherence

**Figure 4:**
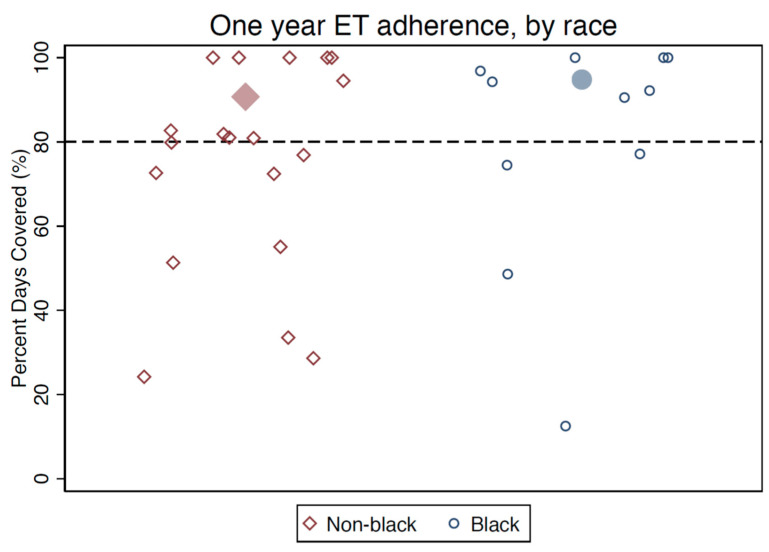
Post-intervention medication adherence by MEMS data (percentage of days covered). Note: Larger filled markers represent group means.

Using MEMS data (a secondary outcome measure), the mean PDC over the 12-month period was well above 80% of the days taking medication for both Black and non-Black participants. Overall, roughly two-thirds of participants were adherent using a threshold of 80% PDC over the first year following enrollment, including 95% of Black participants and 57% of non-Black participants (Figure 4). When participants were asked to self-report medication-taking behavior in the previous 14 days, 88% of women reported taking at least 12/14 doses (>80% adherence), including 100% of Black women and 81% of non-Black women (Table 2). Participants reporting non-adherence on the self-report measure were also generally non-adherent by MEMS; however, 31% of those who were adherent over the past two weeks by self-report did not achieve the threshold for 80% adherence by MEMS, for an overall measure concordance of 73% (Table 2).

## 4. Conclusions

Our study demonstrates the acceptability and early promise of effectiveness of a novel MI counseling-based intervention to promote ET adherence in diverse populations, particularly among Black women. Black women in our study were numerically more likely to be satisfied with the intervention, and to think it helped them, and Black women were numerically more likely to be adherent by both MEMs and self-report, in contrast to earlier observational data, suggesting promise for closing racial gaps in adherence. Optimal ET adherence is essential for the prevention of breast cancer recurrence and breast cancer mortality, and it may be an important lever in reducing race-based disparities in breast cancer survival among those with HR+ disease. With a large majority of patients completing at least three sessions of counseling intervention and completing both MEMS and self-report-based assessments at 12 months, this pilot intervention study provides some signal that the further development and testing of MI counseling-based support for diverse survivors of breast cancer are feasible and promising future directions for research to close racial gaps in ET adherence and to formally evaluate the impact of intervention upon racial disparities in outcomes of HR+ breast cancer.

Few interventions designed to deliver oral anti-cancer medication support have been tested to date, and those that exist have had limited success [23,24,25,26] or are in the early stages of development [23,27,28,29]. Some evidence suggests that mindfulness-based stress reduction and relaxation training can promote long-term adherence to ET [30]. Patel and colleagues recently tested the effects of an outpatient pharmacy team-led intervention that reduced treatment delays in medication initiation, but did not directly assess longer-term adherence [31]. Non-counseling-based interventions have leveraged text message services for reminders, educational support, and electronic medication monitoring and feedback [32,33], but have had mixed results [34]. Other multicomponent intervention packages using intervention mapping and other stakeholder-engaged approaches have been developed and are being further evaluated [18,35]. In light of the existing gaps in the literature, our study suggests that MI counseling is a reasonable potential intervention that can address multiple barriers simultaneously and facilitate person-specific goal setting and problem solving among diverse people with cancer.

MI counseling requires time invested by participants. We observed that a small proportion of consented patients (4/46) decided not to initiate the intervention, and although retention and session completion among those initiating were generally high, some additional patients who started the intervention (7/42, 17%) did not remain engaged throughout the entire study. Attrition rates for MI and other intensive behavioral interventions in cancer are approximately 25% on average with considerable variation, so our intervention is on par with similar programs, although further work is underway to understand the drivers of loss to follow-up and whether these vary by race or age. As one lesson learned from this pilot study, we modified the recruitment strategy of a next-step randomized trial to identify women after the date of ET initiation to enable a better fit of the intervention for patients who have already decided to take ET.

With regard to our secondary measures of adherence, we are encouraged by the generally strong adherence patterns post-intervention, particularly in Black women. In prior work by our group using the same 14-day self-report adherence measure among a diverse sample of women in the Carolina Breast Cancer Study (CBCS), ET adherence at two years post-diagnosis among non-Black women was 84% and among Black women was 76%. While this pilot trial was not designed to directly compare the adherence of participants to the prior CBCS sample, due to a small sample and the inability to statistically test effect modification by race, the numerically higher adherence reported in the current study (88% among non-Black and 100% among Black women) and the relatively stronger adherence among Black women post-intervention are a hopeful signal. We hypothesize that this intervention may have been particularly well-received among Black women because of its person-centric and supportive design, which focused on understanding the barriers in a non-judgmental way, respecting patients’ own lived experiences, resilience, and strengths and leveraging those as support strategies, and providing a counseling venue for problem solving and sharing, which Black women overwhelmingly indicated that they appreciated.

Several limitations accompany this work. First, our study was limited to fewer than 50 participants within central North Carolina who self-selected to enroll in a study focused on ET support; as such, their experiences may not be representative of patients elsewhere or of those who are not interested in such an intervention. Second, our sample size precluded rigorous statistical testing of some of the more interesting observed trends in the data; nevertheless, this pilot study was intended to primarily demonstrate feasibility and acceptability and be hypothesis-generating and has provided preliminary evidence to support a larger trial, in which we are better able to answer these statistical questions. Future studies should also examine broader determinants of ET adherence through robust statistical analyses in larger samples of women from diverse social and economic backgrounds. Third, medication adherence measurement remains challenging in this and other studies due to the inability to directly observe medication-taking behavior in the real world on a continuous basis with minimal patient burden. While we attempted to address this limitation by capturing both the MEMS and self-reported adherence data, intermittent self-report by short interval recall is inherently different than MEMS observation over a longer continuous period, and it is possible that the differences between self-report and MEMS adherence were due to different recall periods rather than a true over-estimation by the patient. The over-estimation of adherence by a two-week self-report compared to a full-year observation by MEMS caps, while consistent with prior findings from non-cancer settings comparing MEMS to self-report [36], is a significant confirmatory finding for cancer populations and highlights the importance of using alternative methods and time periods of adherence measurement in interventional work. Work is underway to test effectiveness more rigorously in an ongoing trial funded by the National Cancer Institute in the Alliance Clinical Trials for Oncology network [37]. In this next step of research, larger sample sizes and serial self-report measurement over longer time periods will enable a better understanding of the relationship between these two measurement approaches in this clinical setting.

In conclusion, ET is a critical component of breast cancer treatment that can be taken for ten years and affects recurrence and survival, and our own data have shown that Black women, in particular, experience significant barriers to adherence that decrease medication taking and warrant intervention [1,2]. In this study, we developed and pilot-tested a behavioral MI counseling intervention designed to improve ET use overall and reduce racial disparities among women with HR+ breast cancer. We designed the GETSET (Guiding Endocrine Therapy Success with Empowerment and Teamwork) intervention with substantive input from ET patients and breast cancer providers, and results indicate that this intervention is feasible, acceptable, and shows early promise for effectiveness in enhancing ET adherence in diverse patient populations that need more active and ongoing support.

## Figures and Tables

**Table 1:** GETSET participant characteristics by race.

	Overall	Black	Non-Black
**N**	35 (100%)	13 (37%)	22 (63%)
**Age, mean (Standard Deviation)**	54.8 (10.7)	55.8 (12.9)	54.2 (9.6)
**Marital Status**			
Never married	7 (20%)	7 (54%)	0 (0%)
Married, or living with a partner	24 (69%)	3 (23%)	21 (95%)
Separated or divorced	4 (11%)	3 (23%)	1 (5%)
**Any dependents in household**	18 (51%)	5 (38%)	13 (59%)
**Educational Attainment**			
High school graduate (or GED)	3 (9%)	2 (15%)	1 (5%)
Technical school/some 4-year college	6 (17%)	3 (23%)	3 (14%)
4-year college graduate	18 (51%)	4 (31%)	14 (64%)
Post-graduate/professional degree	8 (23%)	4 (31%)	4 (18%)
**Annual Household Income**			
Less than USD 20,000	4 (11%)	4 (31%)	0 (0%)
USD 20,000 to USD 49,999	4 (11%)	3 (23%)	1 (5%)
USD 50,000 to USD 99,999	8 (23%)	2 (15%)	6 (27%)
USD 100,000+	14 (40%)	3 (23%)	11 (50%)
I prefer not to answer	5 (14%)	1 (8%)	4 (18%)
**Insurance Type**			
Uninsured	2 (6%)	1 (8%)	1 (5%)
Private	26 (74%)	8 (62%)	18 (82%)
Medicaid	6 (17%)	3 (23%)	3 (14%)
Medicare	1 (3%)	1 (8%)	0 (0%)

**Table 2:** Comparison of MEMS cap to self-report adherence data by race.

	Overall	Non-Black	Black
Adherent by Self Report	28/32 (88%)	17/21 (81%)	11/11 (100%)
Adherence by MEMS (% with > 80% PDC)	21/33 (64%)	12/21 (57%)	9/12 (75%)
Concordance of Self-Report and MEMS	73%	75%	70%

Notes: PDC, proportion of days covered; MEMS cap refers to the medication electronic monitoring system cap used to track open/close events for medication adherence.

## Data Availability

Informed consent was obtained for all subjects involved in the study. De-identified data are available from the corresponding author upon request.

## Appendix A

**Table A1:** Motivational interviewing counseling session content and activities.

Session #	Session Topic	Goal/Activities
**1**	“Welcome to GET SET”	Discuss background/goals of study. Identify ET-related knowledge. Discuss basic educational information about endocrine therapy.
	Values	Establish participant values. Complete “What is Important to You” activity.
	Motivators and ET	Establish participant motivators. Complete “What Motivates You” activity. Discuss established motivators in relation to adherence to ET.
	Barriers/Facilitators to ET adherence	Establish barriers and facilitators at baseline. Complete barriers/facilitators activity. Make a plan and set goals for overcoming barriers.
	Identify Support Person(s)	Discuss importance of support person(s). Establish support person(s).
	Review goals/plans	Review session. Review goals and plans established.
**2–5**	Rapport building	Brief check-in.
	Review previous session goals/plans	Review of Session 1 activities/topics. Review of goals/plans for overcoming barriers. Review of established support person(s).
	Select topic	Participant selection of topic. Example topic: “feeling stressed out”.
	Establish barriers and facilitators	Describe problem/topic. Discuss reason for selecting topic. Establish barriers and facilitators associated with topic. Example problem/barrier: “thinking about the cancer makes me stressed”.
	Make a plan	Elicit potential actions for overcoming problem/barrier. Discuss pros/cons of changing behavior. Example action to overcome: “take more time for myself”. Example plan: “schedule at least 1 h each week to do something by myself”.
	Establish and discuss importance/confidence	Discuss participant motivation to change. Discuss participant self-efficacy in changing behavior.
	Discuss coping strategies	Discuss previous general coping strategies. Elicit potential coping strategies. Complete “Dealing with Setbacks and Coping” activity. Example coping strategy: “talk to my support person/people about my thoughts and struggles”.
	Identify/Review support person(s)	Review previously identified support person(s). Identify/elicit new support person(s), if applicable.
	Goal setting	Identify specific goals to complete prior to next session. Example goal: “next week, I will plan to walk for 1 h with my support person”.
	Review session	Review session, plan, and established goals. Schedule next session.

**Table A2:** GETSET feasibility and acceptability outcomes by race.

	Overall	Non-Black	Black
**Feasibility at 12 months**	(N = 35)	(N = 22)	(N = 13)
Completed 3/5 sessions	34 (97%)	22 (100%)	12 (92%)
Completed 4/5 sessions	29 (83%)	19 (86%)	10 (77%)
Completed 5/5 sessions	19 (45%)	12 (41%)	7 (54%)
Successfully used MEMS	33 (94%)	21 (95%)	12 (92%)
**Acceptability at 12 months**	(N = 32)	(N = 21)	(N = 11)
**Satisfied with counseling overall**	**Overall**	**Non-Black**	**Black**
Agree/Strongly Agree	28 (88%)	18 (86%)	10 (91%)
Neither Agree nor Disagree	3 (9%)	2 (10%)	1 (9%)
Disagree/Strongly Disagree	1 (3%)	1 (5%)	0 (0%)
**Participating in GETSET made it easier for me to stay on my meds**			
Agree/Strongly Agree	21 (66%)	11 (52%)	10 (91%)
Neither Agree nor Disagree	8 (25%)	7 (33%)	1 (9%)
Disagree/Strongly Disagree	2 (6%)	2 (10%)	0 (0%)
**I felt GETSET was a good fit for me**			
Agree/Strongly Agree	27 (84%)	17 (81%)	10 (91%)
Neither Agree nor Disagree	4 (12.5%)	3 (14%)	1 (9%)
Disagree/Strongly Disagree	1 (3%)	1 (5%)	0 (0%)
**The MEMS Cap was easy to use**			
Agree/Strongly Agree	26 (84%)	17 (85%)	9 (82%)
Neither Agree nor Disagree	2 (10%)	1 (5%)	2 (18%)
Disagree/Strongly Disagree	2 (6%)	2 (10%)	0 (0%)
**The GETSET workbook was useful**			
Agree/Strongly Agree	20 (65%)	11 (52%)	9 (82%)
Neither Agree nor Disagree	29(29%)	7 (33%)	2 (18%)
Disagree/Strongly Disagree	3 (10%)	3 (14%)	0 (0%)
**Scheduling my GETSET counseling sessions was convenient and easy**			
Agree/Strongly Agree	28 (77%)	18 (86%)	8 (73%)
Neither Agree nor Disagree	4 (13%)	2 (10%)	2 (18%)
Disagree/Strongly Disagree	2 (6%)	1 (5%)	1 (9%)
**I wish there had been more counseling sessions**			
Agree/Strongly Agree	8 (25%)	4 (19%)	4 (36%)
Neither Agree nor Disagree	10 (31%)	5 (24%)	5 (45%)
Disagree/Strongly Disagree	14 (44%)	12 (57%)	2 (18%)
**I wish there had been fewer counseling sessions**			
Agree/Strongly Agree	4 (12%)	3 (14%)	1 (9%)
Neither Agree nor Disagree	18 (56%)	12 (57%)	6 (55%)
Disagree/Strongly Disagree	10 (31%)	6 (29%)	4 (36%)

Note: Percentages may not add to 100 due to rounding.
